# Obstructive Ureterocele Stone Mimicking an Intravesical Stone

**DOI:** 10.5334/jbr-btr.1433

**Published:** 2018-01-03

**Authors:** Sammy Tawk, Etienne Danse

**Affiliations:** 1 Cliniques universitaires Saint-Luc, BE

**Keywords:** Ureterocele, Hydronephrosis, Ultrasound, Obstructive stone, CT scan, Trick

A 69-year-old man presented to the emergency department for sudden onset of severe right flank pain radiating to the groin. Lab tests were all negative. Right renal colic was clinically suspected and a non-contrast abdominal computed tomography (CT) was performed to rule out an obstructive ureteral stone or other intra-abdominal abnormalities.

**Figure 1:**
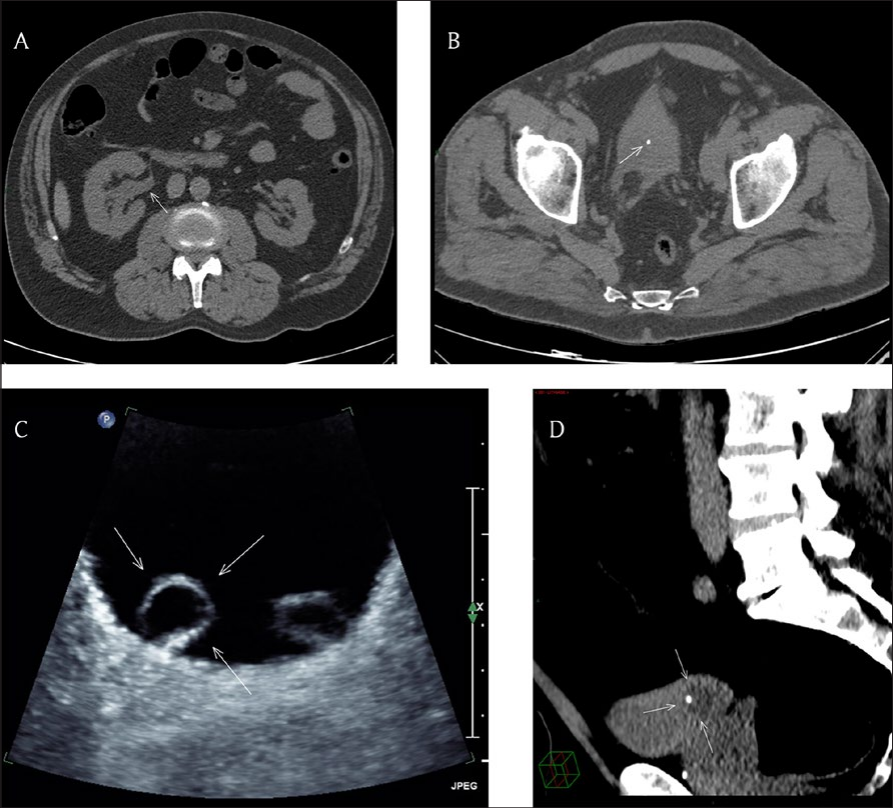
**(A)** Axial CT scan image (non-contrast) at the level of the abdomen showing right uretero-pyelo-calyceal dilatation (arrow). **(B)** Axial CT scan image (non-contrast) at the level of the pelvis showing a 3.6 mm hyperdense stone in an almost empty bladder (arrow). **(C)** Axial US image of the bladder showing bilateral ureterocele, more prominent at right (arrows). **(D)** Reformatted sagittal CT scan (non-contrast) image with narrow window width of the pelvis showing the urine filled right ureterocele with the stone located inside (arrows).

CT scan showed a right uretero-pyelo-calyceal dilatation (Figure 1A, arrow) with the presence of a 3.6 mm hyperdense stone in an almost empty bladder (Figure 1B, arrow). A non-obstructive left renal stone was also present. The rest of the exam was unremarkable. The patient was discharged home after he received the post-hospital discharge protocol specific to his case.

Three weeks later, as part of the regular follow-up, an abdominal ultrasound (US) was performed that showed resolution of the right hydronephrosis with evidence of bilateral ureterocele [1], more prominent at right (Figure 1C, arrows). No urinary tract stone was seen.

Retrospectively, a review of the multiplanar reformatted CT scan images with narrow window width showed a subtle density difference between the empty bladder and the urine-filled right ureterocele with the stone located actually in the ureterocele (Figure 1D, arrows) and not freely in the bladder.
